# Strongyloides

**DOI:** 10.1017/S0031182016001773

**Published:** 2016-11-21

**Authors:** MARK VINEY

**Affiliations:** School of Biological Sciences, University of Bristol, Bristol BS8 1TQ, UK

The *Strongyloides* genus of nematodes are common parasites of terrestrial vertebrates, and ones that have a fascinating biology. In humans, they are one of the soil-transmitted helminthiases (STH) a WHO-recognized neglected tropical disease (NTD). But, compared with the other STH parasites – *Ascaris*, hookworms and *Trichuris* – *Strongyloides* is the poor relative, arguably itself rather neglected.

*Strongyloides* was discovered 140 years ago in French troops returning from modern-day Vietnam. After its discovery, and following some great taxonomic complexity, its name was settled, coming from the Greek words ‘strongylos’ meaning ‘round’, and ‘eidos’ meaning ‘similar’, together intending to show that *Strongyloides* was close to the genus *Strongylus* (Grove, 1989). With today's perspective this is a sadly unimaginative name, but perhaps slightly better than *Strongylus* itself. Notwithstanding, the intervening century and a half has now given us an unprecedented understanding of *Strongyloides* biology, which is brought together in this volume.

Parasitologists of all flavours are (rightly) fascinated with life cycles, but this is perhaps particularly appropriate with *Strongyloides*. Here this life cycle is described, to save it being repeated in every paper contributing to this volume. The following description is largely based on the life cycle of *Strongyloides ratti* in rats, simply because this species has been most thoroughly studied, and because its life cycle is generally representative of different *Strongyloides* species.

**Fig. 1.**
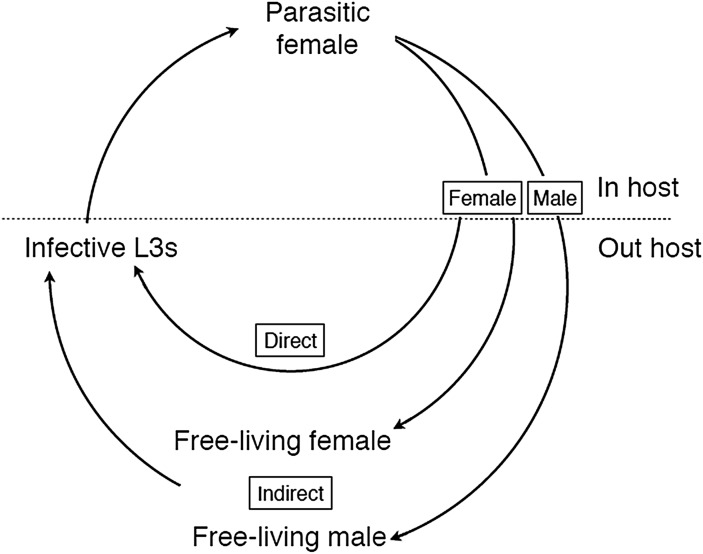
The life cycle of *Strongyloides* showing the obligate female-only parasitic generation and, outside of the host, the two modes of development – direct larval development or facultative, indirect development via free-living adults. Larval stages are omitted for clarity.

**Fig. 2.**
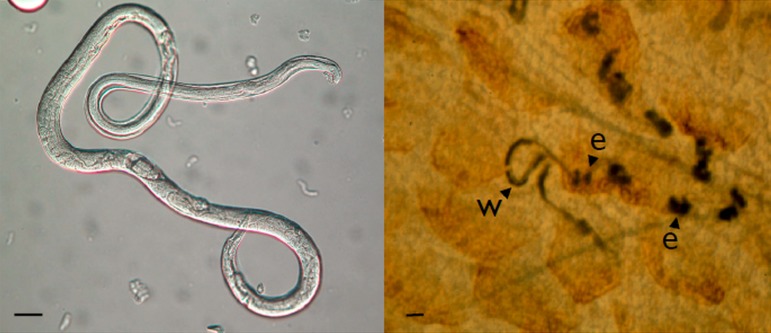
The parasitic female of *Strongyloides ratti*, free of host tissue (left, bar = 30 *µ*m) and embedded in host mucosal tissue (right, bar = 100 *µ*m), showing the worm (w) and egg clumps (e); Viney and Lok (2015).

**Fig. 3.**
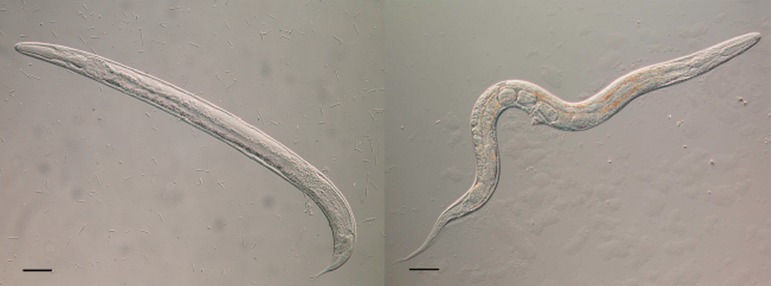
A free-living male (left) and free-living female (right) of *Strongyloides ratti*. Both bars = 50 *µ*m; Viney and Lok (2015).

Compared with most other parasitic nematodes, the *Strongyloides* life cycle is unusual because it has two adult generations – one in the host and one outside (Fig. 1). The parasitic adult generation is female-only and these reproduce by parthenogenesis, which is genetically mitotic (Fig. 2). The parasitic females produce eggs that are, genetically, male and female. These eggs, or the L1s that hatch from the eggs, pass out of the host in its feces (which stage is passed being a species-specific character), where the larvae then grow, develop and moult. Males and females have different developmental fates. Male eggs (or larvae) moult through four larval stages (L1–L4) and then into free-living adult male worms (Fig. 3). The female eggs (or larvae) have a developmental choice. In one option, they can develop analogously to males (moulting through four larval stages) finally moulting into free-living adult female worms (Fig. 3). Alternatively the female larvae can moult through three larval stages into third-stage larvae (L3s), which are infectious to a new host.

The free-living males and females sexually reproduce, and the female then lays eggs. These hatch and the resulting larvae moult via an L2 stage into third-stage larvae (as above). Crucially, there is only a single free-living adult generation and all the free-living females’ progeny develop into host infective L3s. [This contrasts with the close relative, *Parastrongyloides*, where there can be multiple free-living adult generations (Grant *et al.* 2006)].

Because the aim of this free-living life cycle is to produce infective third-stage larvae, the two developmental routes to producing these are known as direct (or homogonic) and indirect (or heterogonic) (Fig. 1). Infective larvae are developmentally arrested, and only reinitiate development when they successfully penetrate the skin of a suitable host. These larvae migrate through the host, moulting via an L4 stage before settling in the host gut where they moult into parasitic females, and the cycle is then complete. One notable species-specific difference in this life cycle is for the parasite of humans, *Strongyloides stercoralis*, where infective L3s can precociously develop within the host causing internal auto-infection, which makes human infections chronic.

The two adult generations are quite distinct. Apart from one being parasitic and parthenogenetic and the other being free-living and sexual, they differ morphologically. This is most easily seen with the oesophageal morphology, where the parasitic females have a filariform-style oesophagus that occupies about a third of their body length, whereas the free-living adults’ is rhabditiform and about 10% of their body length. All of the free-living larval stages also have a rhabditiform-style oesophagus, except for the infective L3s, which is filariform, as is the parasitic females’ into which they will develop. The free-living adult stages are especially experimentally amenable, and have been instrumental in genetic analysis of *Strongyloides* and in developing transgenesis.

*Strongyloides* first came to the attention of science because of infection of humans. Here Tom Nutman brings up-to-date our understanding of human infection (Nutman, 2016). Of the 30–100 million infections most are asymptomatic, but people with an altered immune status – particularly because of the administration of corticosteroids or HTLV-1 infection – can have more intense infections, ultimately leading to hyperinfection, which can be fatal if unrecognized. *Strongyloides* infection is also well known among livestock, and this is reviewed by Stig Thamsborg and colleagues (Thamsborg *et al.* 2016). In general, *Strongyloides* is now of relatively little importance in livestock, due to regular anthelminthic treatment and high husbandry standards. But where disease does occur it tends to be concentrated in young animals. The taxonomy of the *Strongyloides* species infecting livestock is poorly known, and much work remains to be undertaken in this area. Of particular interest is the status of *S. stercoralis*, which is described as a parasite both of dogs and of people. However, whether these reports represent one species truly being shared by dogs and humans has not been rigorously addressed. The possibility that human strongyloidiasis is a zoonosis from dogs warrants rapid investigation.

The parasites of rats *S. ratti* and *Strongyloides venezuelensis* have been a mainstay of *Strongyloides* research, which has led to the very considerable detail with which we understand the *Strongyloides* life cycle, and this is reviewed here by Mark Viney and Taisei Kikuchi (Viney and Kikuchi, 2016). *Strongyloides ratti* and *S. venezuelensis* are common parasites, with *S. ratti* being ubiquitous, while *S. venezuelensis* is restricted to warmer climates. They are both excellent laboratory models. It is particularly intriguing that rats are host to two *Strongyloides* species – likely representing two independent transitions to parasitism of rats – while there is no known species that naturally infects mice (and *S. ratti* and *S. venezuelensis* are poor parasites of mice) (Viney and Kikuchi, 2016).

The interaction of *Strongyloides* with its host immune response is key to understanding the harm that it can cause to hosts, and the immunobiology of *Strongyloides* is reviewed here by Minka Breloer and David Abraham (Breloer and Abraham, 2016). In common with other helminths a Th2 response dominates host anti-*Strongyloides* responses. But, this results in different, site-specific effects acting against either migrating larvae within the host tissues or against parasitic adults in the host gut. The effectors acting against parasitic adults are mast cells [activated by the cytokines interleukin (IL)-3 and IL-9] and anti-*Strongyloides* immunoglobulin E (IgE) and IgG responses (Breloer and Abraham, 2016). The developmental choices of the *Strongyloides* life cycle are in part controlled by the host immune response (Viney and Kikuchi, 2016), and understanding what aspects of the host immune response *Strongyloides* sense, and how they sense it, to make these life cycle decisions is unknown, and something that deserves to be investigated. The phenomena of helminth parasites sensing and interacting with their host immune response to make life history decisions is likely to be common among many, if not all, helminths (e.g. Babayan *et al.* 2010). To date there has been rather little examination of these phenomena, though clearly this is a fascinating research area for the future.

The genetics of the *Strongyloides* life cycle have also been of enduring interest – and confusion – to researchers, and the current, sophisticated understanding of *Strongyloides*’ genetics is reviewed here by Adrian Streit (Streit, 2016). The most detailed genetic analyses have been for *S. ratti* and *Strongyloides papillosus*, which also highlights the interesting species-specific differences, since these species represent two sub-clades within the genus. Thus, in *S. ratti* the haploid chromosome numbers is 3, consisting of two autosomes and an X chromosome, and sex is determined by a female/male, XX/XO system. In *S. papillosus* the diploid chromosome numbers is 4, because the X chromosome has ancestrally become fused to an autosome, thereby generating one long chromosome and one short chromosome. In males sex is determined by diminution of the X-chromosome-equivalent region of the fused chromosome (Streit, 2016). In effect these two species have different mechanisms of changing the dose of the X chromosomes to control sex, with these different methods necessitated by the different chromosomal arrangement of these species.

2016 was a key year for *Strongyloides* because the genomes of four *Strongyloides* species were sequenced, and these results were used to begin to understand *Strongyloides*’ genomic adaptations to parasitism. Here Vicky Hunt and colleagues review this (Hunt *et al.* 2016*a*). This genome sequencing work showed that *Strongyloides* has a compact genome, indeed almost the smallest known nematode genome. Comparison of the *Strongyloides* genome with that of close relatives – the facultative parasite *Parastrongyloides trichosuri* and the free-living species *Rhabditophanes* – identified the gene families that expanded as the parasitic lifestyle evolved (Hunt *et al*. 2016*b*). Here the *Strongyloides* life cycle (Fig. 1) was also exploited to understand *Strongyloides*’ adaptations to parasitism, by comparing the parasitic and free-living adult females, to identify the genes and proteins specified for the parasitic female stage. Together this has given an unrivalled view of the genetic basis of *Strongyloides*’ parasitic lifestyle. In this volume, each of the major parasitism-associated gene families – those coding for the astacin metallopeptidases, aspartic proteases, SCP/TAPs-containing proteins, acetycholinesterases, transthyretin-like proteins, prolyl oligopeptidases – are considered in more detail to ask what parasitism-specific roles these gene products might play (Hunt *et al.* 2016*a*).

Key to making headway in understanding how genes and their products allow and facilitate the parasitic lifestyle are methods for transgenic analysis. While such methods have existed for the model nematode *Caenorhabditis elegans* for 30 years, achieving this for parasitic nematodes has proved much harder. Work with *Strongyloides* has led the way, driven by James Lok, who here reviews this work (Lok *et al*. 2016). Transgenesis of *Strongyloides* has been considerably harder to achieve than in *C. elegans*, both because *Strongyloides* silences introduced constructs, and because it is rather fragile when being injected. But these methods are now well established. Methods for genome editing are clearly moving apace, especially with CRISPR/Cas9 methods: here is presented the first proof-of-principle of this in *Strongyloides* (Lok *et al*. 2016).

All told, in under a century and a half we have moved from discovering a curious worm infecting soldiers in the far East, then finding other species in a wide variety of hosts, eventually unpicking its complex life cycle, whose genetics and immunological interactions with its hosts we now understand. The recent genome analyses of this parasite now bring us full circle as we are poised to ask with a growing armamentarium of tools – what does it take to be a parasitic nematode?
